# Decolonising global health evaluation: Synthesis from a scoping review

**DOI:** 10.1371/journal.pgph.0000306

**Published:** 2022-11-16

**Authors:** Ichhya Pant, Sonal Khosla, Jasmine Tenpa Lama, Vidhya Shanker, Mohammed AlKhaldi, Aisha El-Basuoni, Beth Michel, Khalil Bitar, Ifeanyi McWilliams Nsofor

**Affiliations:** 1 Department of Prevention and Community Health, George Washington University School of Public Health, Washington, DC, United States of America; 2 Independent Evaluation Scholar and Practitioner, Vancouver, British Columbia, Canada; 3 Department of Pharmacology & Experimental Therapeutics, Thomas Jefferson University, Philadelphia, PA, United States of America; 4 Interdependent Evaluation Scholar and Practitioner, Minneapolis, Minnesota, United States of America; 5 Faculty of Medicine and Health Sciences, McGill University Health Center (MUHC), McGill University, Montreal, QC, Canada; 6 Faculty of Medicine and Health Sciences, School of Physical and Occupational Therapy (SPOT), McGill University, Montreal, QC, Canada; 7 Canadian Institutes of Health Research (CIHR), Health Systems Impact Fellowship, Ottawa, Canada; 8 Department of Environmental Health Sciences, Faculty of Communication, Arts and Sciences, Canadian University Dubai (CUD), Dubai, United Arab Emirates; 9 University of Basel, Swiss Tropical and Public Health Institute (Swiss TPH), Basel, Switzerland; 10 Council on Health Research for Development (COHRED), Research Fairness Initiative, Geneva, Switzerland; 11 An-Najah University, North Gaza, Gaza Strip, Palestine; 12 Indigenous Evaluator and Public Health Practitioner, Office of Undergraduate Admissions, Emory University, Atlanta, GA, United States of America; 13 Khalil Bitar, Wyss Academy for Nature, University of Bern, Bern, Switzerland; 14 Nigeria Health Watch, Abuja, Nigeria; Université de Sherbrooke: Universite de Sherbrooke, CANADA

## Abstract

As decolonisation awareness and activism amplifies in the mainstream masses and within academic realms across a variety of fields, the time is right to converge parallel movements to decolonise the fields of global health and evaluation by restructuring relations of dependency and domination reified through the “foreign gaze”^1^ or “white gaze.” We conducted a review of relevant records with the following inclusion criteria–they define or advocate for the decolonisation of global health evaluation or explicate methods, policies or interventions to decolonise global health evaluation published by advocates of the decolonisation movement from both fields. These records were derived following a systematic article search by the lead autthor on Google, Google Scholar, NewsBank, and PubMed using the following keywords: “decolonising” and “global health,” “evaluation,” or “global health evaluation” replicating a digital search strategy utilized by scoping reviews across a variety of topics. Because the topic of interest is nascent and still emerging, the date range was not restricted. The lead author screened abstracts retrieved from the search. In total, 57 records, ranging in publication date from 1994 to 2020, were selected and charted for this review. We reviewed these records to identify socio-ecological factors that influence the decolonisation of global health evaluation, such as decolonising minds; reorienting funders and reforming funding mechanisms; and investing in sustainable capacity exchange. In doing so, we reflected on our positionality as well as our internalisation and potential reinforcement of colonial relations in the process of reporting our results. In the context of turmoil and transition due to the COVID-19 pandemic, our scoping review offers a starting point to embark on a journey first to transform and decolonise global health evaluation and then to achieve the greater goal of equity and justice.

## Introduction

The concept of the “foreign gaze” [1] or “white gaze” [2] of development assumes whiteness as the primary reference of power, prestige, and progress across the world. The prevailing foreign or white gaze of development measures the political, socio-economic, and cultural processes of the Global South against the expectations of Northern saviourism and standard of Northern supremacy.The same applies to scientific scholarship and practice. Among many sectors, two fields that the foreign or white gaze has plagued are global health and evaluation [3]. While the fields of global health and evaluation have called out the importance of cultural competence (see Mews, Schuster & Vadja, et al., 2018 [4] to gain a nuanced and thorough understanding of the influence of cultural competence on global health evaluation), their failure to integrate curricula that acknowledge and repair damage from both fields’ colonial roots is telling [5, 6]. Emerging scholars are now explicitly naming the “depoliticized, un-critical, and ahistorical” disciplinary lens maintained within these fields, whose obliviousness to unequal economic, social, and power relations steeped in historical and ongoing injustice feels wilful to those who are disadvantaged by those relations [7].

### Colonial legacy of Global Health

Despite the rising prominence and proliferation of Global Health efforts since World War II, Global Health trudges forward without a universally agreed upon established definition [8, 9]. Of the many proposed definitions for Global Health, this review will adopt the definitional lens offered by Beaglehole and Bonita (2010) [8] which centres transnational collaboration, research, and action to promote health for all. Collaborative co-creation of an evidence-base and its application through actions such as interventions, policies, or other constructive public health strategies to improve health equity and ensure health for all aligns with the ethos guiding this review. While all definitions of Global Health signal transnational perspectives, a phrase that implies lateral rather than colonial relationships, they are remiss in their omission of the health status of approximately four hundred million indigenous people. Drawing from postcolonial frameworks, we consider Indigenous Health an integral component of Global Health. Additionally, disparities in the health outcomes between racially otherized peoples and whites worldwide can largely be attributed to the detrimental effects of colonization and enslavement, exacerbated by the colonial legacy, capitalist economy and present propensities of Global Health as a field [10]. Drawing from critical theories more generally, we similarly centre colonized and enslaved peoples as a whole.

The official narrative of the history of the field of global health proclaims that its origins lie in colonial efforts to protect colonial settlers and administrators from acquiring infectious diseases prevalent within colonies in the tropics. Perhaps a decolonised perspective would revise this historical account by offering a counter-narrative—for example, indigenous efforts to protect the peoples of Turtle Island (now called North America) from infectious diseases that were prevalent among colonisers precedes contemporary global health [11]. While territorial colonialism might have ended decades ago in many but not all parts of the world, the colonisation of minds, cultures, politics, and economies continues and reparations are yet to be realised [11, 12]. Over the last few decades, global health scholars have called to improve health justice universally. A recently developed ethical framework titled *Research for Health* Justice was developed that provides guidance on meaningfully promoting global health equity through metrics such as, what research populations and questions ought to be selected, what research capacity strengthening ought to be performed and what post-study benefits ought to be provided [13]. Despite the broadened scope of global health justice, colonial knowledge and practices still dictate policies and decision making in the field. Those from the Global South are largely relegated to token positions—for example, primarily as enumerators and implementation, research or evaluation partners and rarely as principal investigators—in global health discussions rather than inhabiting powerful roles in policy and decision making [12]. The current global health ecosystem and its governance structure are not equipped to address contextual disadvantage in the Global South as a determinant of health [14]. As it became evident during the COVID-19 pandemic, many strategies implemented by Western structures such as lockdown and social distancing are impossible privileges that communities living in slums in the Global South cannot avail, triggering a high prevalence of diseases among such communities [15]. These points suggest that decolonising global health involves much more than merely adding seats for members of the Global South at the proverbial table. Diverse composition of authority does not automatically translate into changed values or reformed structures. We suspect that decolonizing the field of global health may require transforming the way in which we think about global health overall, understand the “winners” and “losers” of systems of oppression, and demonstrate awareness of who has the final say in decision making. Decolonising the field of global health likely further requires redefining its purpose, reimagining the system, and reformulating its policies and rules accordingly.

### Colonial influence in the field of evaluation

As donor assistance in the form of Global Health investments has increased in the past two decades, the Global Health community is embracing the evaluation of Global Health efforts with resolve [16]. There is consensus on the need to determine the effectiveness of Global Health investments to enhance evidence building, support decision making and capacity building [17]. The United States Agency for International Development (USAID) frames evaluation of Global Health programs as a systematic endeavour which generates insight on programmatic processes and outcomes to enhance accountability, incorporate learning to improve developmental outcomes, and guide strategic planning of future investments [18].

Not unlike the global health field, evaluation theory, policies, and practices derive from and have historically been dictated by Western frameworks that fail to recognise and that systematically exclude ontologies and epistemologies that are culturally distinct from those of the European Enlightenment [19, 20]. Although the field of evaluation is intended to garner effective interventions while mitigating iatrogenic effects [21], it is grappling with its own colonial legacy and epistemological limitations [22]. Evaluation as a field is embedded in the contested power relations that plague international development as a whole and it has until recently articulated no need to fundamentally transform the power relations in which it is embedded. Among other groups, indigenous peoples and researchers have made entirely clear that they want evaluations that are “of, for, by and with us” and research that does not “plan about us, without us” [23, 24]. Evaluators increasingly voice that evaluations should honour local beliefs, manners, and customs and act with integrity and honesty in their relations with all stakeholders, prioritising the most marginalised groups [25]. Although the field of evaluation considers cultural competence, the field has yet to develop a critical body of literature or guidelines for practice that interrogate understanding of difference and inequality, including its own role therein [20].

### Call to decolonise global health evaluation

As decolonisation awareness and activism amplifies in the mainstream masses and within academic realms across a variety of fields, the time is ripe to converge parallel movements of decolonising global health and decolonising evaluation (hereafter collectively referred to as Global Health Evaluation) by rethinking the existing models and restructuring relations of dependency and domination reified through the foreign or white gaze [1, 2]. Under the backdrop of rising social inequality and injustice, there is an awakening of social consciousness tying limitations in the meaningful advancements of these fields to lack of true diversity with respect to ways of knowing and being [20, 26].

The American Evaluation Association is centering the decolonisation of evaluation in its annual conference in 2022. Similarly, in recent years, there have been innumerous conferences and workshops in the field of global heath to fuel the demand for this subject matter worldwide.

### Aims and objectives

This paper offers a description of the decolonisation of global health evaluation as specified by professionals advocating for decolosniation in their respective fields. Furthermomre, we offer a socio-ecological lens to decolonise global health along with, and by, materially decolonising evaluation.

This paper begins to answer the following research questions: 1) How do the advocates of the decolonisation global health evaluation movement describe the decolonisation of global health evaluation? 2) What socio-ecological factors do advocates of the decolonisation of global health evaluation associate with the decolonisation of global health evaluation? It draws on critical theories, particularly postcolonialism, which raises questions about the power of the gaze in defining the other relative to Europe/ European settler states [27]. To a lesser degree, this study also draws on critical whiteness studies, which names whiteness as the unmarked standard of normativity [28, 29].

## Methods

This scoping reviewutilises Arksey and O’Malley’s (2005) scoping methodology [30] and Bronfenbrenner’s (1979) socio-ecological model [31] as a conceptual framework to synthesise peer-reviewed and grey literature published on topics related to the decolonisation of global health, evaluation, or global health evaluation. Bronfenbrenner’s systems-oriented socio-ecological model [31] theorises that factors at various levels of individuals’ environment shape their development, health, and well-being, both independently and interactively. Our study adapts this conceptual framework to produce a socio-ecological perspective on factors that are associated in the literature with the decolonisation of global health, evaluation, or both specialties collectively.

**Fig 1:**
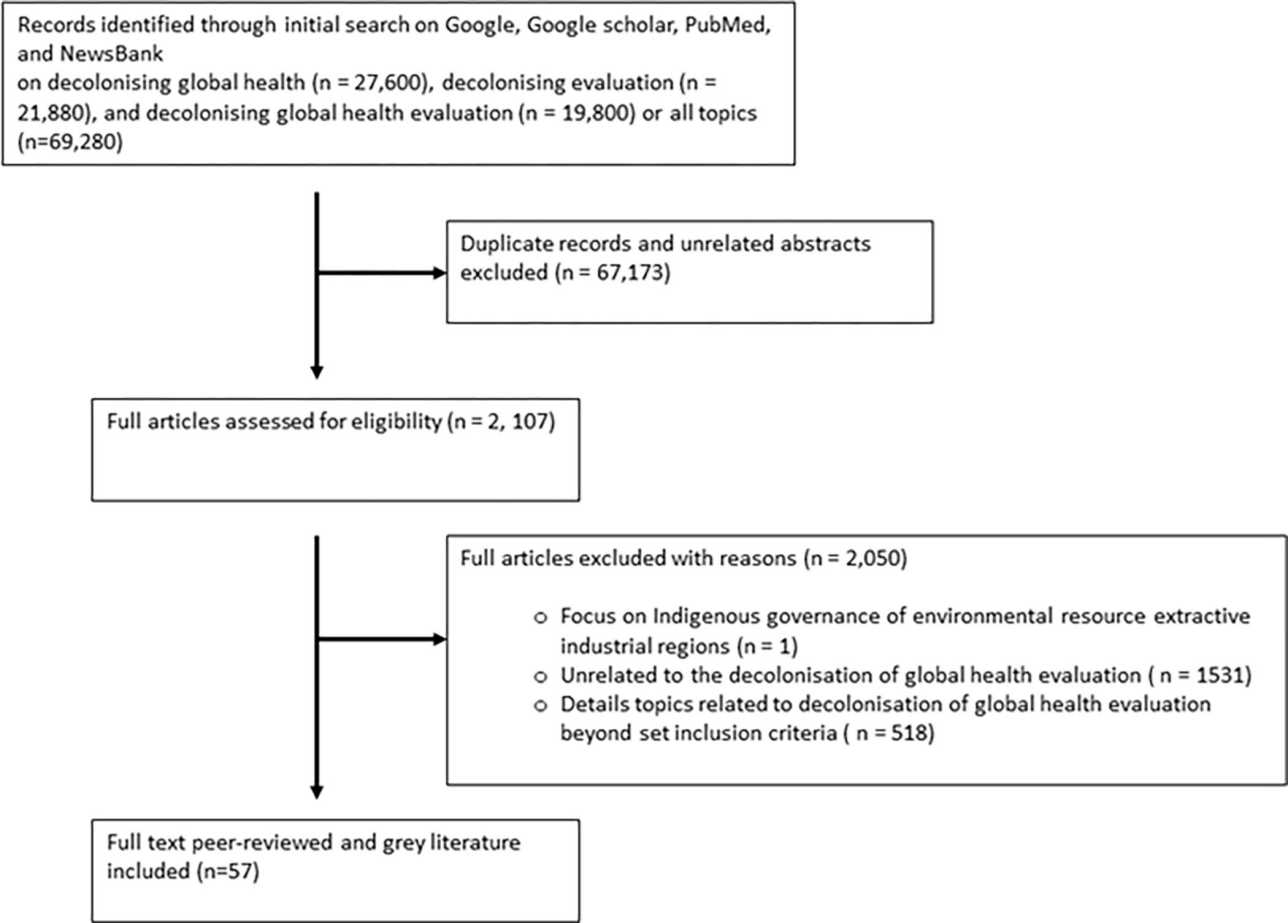
PRISMA flow chart of scoping review (based on Arksey & O’Malley 2005).

A scoping methodology offers rigor, structure, and transparency to synthesise studies that are 1) focused on nascent topics to identify key concepts associated with them, or 2) conducted using disparate methodologies and tools to foster reliability and replicability of study findings [32, 33]. Both apply to the topics of decolonisation of global health and evaluation. Research activities were conducted between September 2020 and January of 2021. Inadequate resources and linguistic capacity within the authorship team limited the scope of selection to records published in English (Fig 1). This scoping review did not warrant institutional ethical approval because it did not involve primary research with human subjects.

### Search strategy and eligibility criteria

The lead author (IP) conducted a systematic article search on Google, Google Scholar, NewsBank, and PubMed using the following keywords: “decolonising” and “global health,” “evaluation,” or “global health evaluation” replicating a digital search strategy utilized by scoping reviews across a variety of topics [32, 33]. Because the topic of interest is nascent and still emerging, the date range was not restricted (see Table 1 for details on full search strategy). These databases were selected to account for and incorporate both grey and scholarly literature in our search strategy. The lead author screened abstracts retrieved from the search. Records were selected if they included one of the following six criteria:

1. a call to decolonise global health evaluation;
2. a conceptualisation of theories or frameworks to decolonise global health evaluation;
3. a definition of decolonisation of global health evaluation;
4. an explication of methods to decolonise global health evaluation;
5. detailed interventions or curricula to decolonise global health evaluation; and
6. a description or demonstration of capacity to decolonise global health evaluation.

**Table 1:** Digital search strategy.

Database/Search Engine	
PubMed	decolonising, global health, evaluation, global health evaluation, decolonising global health evaluation, decolonising global health, decolonising evaluation, decolonising global health evaluation
Google Scholar	decolonising, global health, evaluation, global health evaluation, decolonising global health evaluation, decolonising global health, decolonising evaluation, decolonising global health evaluation
Google	decolonising global health evaluation, decolonising global health, decolonising evaluation, decolonising global health evaluation
NewsBank	decolonising global health evaluation, decolonising global health, decolonising evaluation, decolonising global health evaluation

### Data charting process

The lead researcher (IP) reviewed all selected records (N = 57) and developed, collaboratively with the research team, a structured charting framework which includes descriptive information for all records and emerging themes identified during the preliminary reading of included records (See S1 Table to review our charting template). Then, each research team member charted randomly-assigned records using the structured charting framework. Through this process, relevant data were extracted from records and put into the following categories: article title; number of pages; publisher; geographic location of authors’ affiliated institutions (city, state, country); applicable inclusion criteria (assigned researchers selected one out of the six listed above); definition for decolonising global health, evaluation, or global health evaluation (assigned researchers selected one); methods utilized or proposed; theoretical framework proposed or applied; barriers and facilitators; curricula proposed or applied; role of self-determination, empowerment, or sovereignty; role of cultural, spiritual, holistic, or social justice philosophy and values; role of funding and funders; any legislation, policies, or structural programs mentioned or implemented; role of knowledge generation, mobilization, and mutually beneficial reciprocity; key actors or activists; role of community support, participation, and consent; role of positionality and gaze with respect to the community (insider, outsider, insider-outsider); role of domination and control vis-à-vis liberation or transformation; efforts to build capacity and south-to-south collaborations; role of trust or mistrust; role of solidarity; role of collectivism and inter-connectedness; role of power and privilege; any additional factors/themes unlisted in the charting framework; and general notes.

### Synthesis of results

Following the charting of data, the research team used Bronfenbrenner’s socio-ecological model (1979) [31] to collate and summarise their results in a template developed by the lead researcher. Based on the results summary template, researchers clustered the factors that advocates for decolonisation of global health evaluation who authored the selected records associated with the decolonisation of their respective fields into each level of the socio-ecological model: micro, meso, exo, and macro (see Table 2). The next step in synthesizing our results involved one researcher reviewing the results summaries submitted by all co-authors and developing an initial analytical framework [32–34]. They coded all applicable factors within the initial analytical framework for each record and incorporated new factors into the framework as they emerged. This analytical framework was shared with the entire authorship team and finalized with their feedback. Previously coded records were re-coded using the finalized analytical framework. All remaining records were then coded for applicable themes using the finalized analytical framework. Afterwards, the lead author validated all coded records with a second round of coding followed by a third round of review by research team members for the group of records assigned to them.

**Table 2:** Descriptive characteristics, inclusion criteria, and factors associated with decolonisation by advocates in the field of global health and evaluation within included studies.

Title	Type	Year	Author/s	Authors’ Location	Inclusion Criteria						Socioecological Factors																
											MICRO			MESO					EXO			MACRO					
					CL	CO	DD	EM	DIC	DCA	SD	DIM	IS	RF	ELP	CK	CP	SCE	PC	PG	PP	ES	AM	CSD	CS	DCM	RR
1. Health promotion and the discourse on culture	Peer reviewed Journal Article	1994	Airhihenbuwa, C.	USA					✓							✓	✓		✓		✓	✓		✓	✓		✓
2. Indigenizing evaluation research: How Lakota methodologies are helping “Raise the Tipi” in the Oglala Sioux Nation	Peer reviewed Journal Article	2004	Robertson et al.	USA				✓						✓	✓		✓	✓	✓	✓	✓	✓	✓	✓	✓	✓	
3. Indigenous evaluation can decolonize us	Peer reviewed Journal Article	2006	Deschenie, T.	USA		✓								✓		✓	✓	✓	✓	✓	✓	✓	✓	✓	✓		
4. Appropriate engagement and nutrition education on reserve	Peer reviewed Journal Article	2010	Tobin et al.	Canada		✓					✓		✓				✓	✓	✓	✓	✓	✓	✓				
5. Reframing evaluation: defining an indigenous evaluation framework	Peer reviewed Journal Article	2010	LaFrance et al.	USA				✓								✓						✓					
6. A decolonizing approach to health promotion in Canada: the case of the urban Aboriginal community kitchen garden project	Peer reviewed Journal Article	2010	Mundel et al.	Canada		✓										✓	✓	✓	✓	✓	✓	✓	✓	✓	✓	✓	
7. Decolonize philosophy/being life: the power of discourse in western and Africanist epistemologies of life and the revolution of language in AIDS narratives	Dissertation	2010	Clinton Wills, D.	USA		✓										✓					✓						
8. A framework for decolonization interventions: broadening the focus for improving the health and wellbeing of Indigenous communities	Peer reviewed Journal Article	2010	Guerin, B.	Australia				✓							✓							✓		✓	✓		
9. Beyond informed consent: how is it possible to ethically evaluate Indigenous programs?	Seminar paper	2011	Williams et al.	Australia		✓								✓			✓		✓	✓	✓	✓		✓	✓		
10. Decolonising evaluation in a developing world	Report	2011	Hopson et al.	USA		✓					✓		✓	✓			✓	✓	✓	✓	✓	✓	✓	✓	✓		✓
11. Kaupapa Māori–theory-based evaluation	Peer reviewed Journal Article	2012	Kerr, S.	New Zealand		✓											✓		✓	✓	✓	✓	✓	✓	✓		
12. Decolonisation–a brief history of the word	Book chapter	2012	Betts, R.	USA			✓					✓										✓				✓	
13. Decolonization is not a metaphor	Peer reviewed Journal Article	2019	Tuck et al.	USA			✓													✓	✓	✓		✓	✓		
14. Decolonising evaluation: the necessity of evaluation advisory groups in Indigenous evaluation	Peer reviewed Journal Article	2012	Johnson-Goodstar, K.	USA		✓								✓		✓	✓	✓	✓	✓	✓	✓	✓	✓	✓		
15. Decolonisation of social science research and practice in Latin America	Peer reviewed Journal Article	2013	Meckesheimer, A.	Germany		✓											✓	✓			✓	✓					
16. Across the colonial divide–conversation about evaluation in Indigenous contexts	Peer reviewed Journal Article	2013	Marama Cavino, H.	USA		✓										✓	✓	✓			✓	✓					
17. “Because we have really unique art”: Decolonizing research with Indigenous youth using the arts	Peer reviewed Journal Article	2014	Flicker et al.	Canada				✓				✓					✓	✓	✓	✓	✓	✓	✓	✓	✓		✓
18. Made in Africa Evaluation Concept	Synthesis paper	2015	Chilisa, B.	Botswana		✓								✓		✓	✓	✓	✓	✓	✓	✓	✓	✓	✓	✓	
19. Self-determination and the right to health: Australian Aboriginal community-controlled health services	Peer reviewed Journal Article	2016	Mazel, O.	Australia		✓					✓		✓			✓	✓	✓	✓	✓	✓	✓	✓	✓	✓		
20. Considering the social determinants of equity in Intl Development Evaluation guidance documents	Peer reviewed Journal Article	2016	Robertson, K.	USA			✓							✓	✓	✓	✓	✓	✓	✓	✓	✓	✓	✓	✓		✓
21. A transcultural global systems perspective: in search of Blue Marble Evaluators	Peer reviewed Journal Article	2016	Quinn Patton, M.	USA		✓										✓	✓	✓	✓	✓	✓	✓	✓	✓	✓		✓
22. A cross-cultural evaluation conversation in India: benefits, challenges, and lessons learned	Peer reviewed Journal Article	2016	Al Hudib et al.	India & Canada		✓											✓	✓				✓					
23. Decolonising and indigenizing evaluation practice in Africa: toward Africa relational evaluation approaches	Peer reviewed Journal Article	2016	Chilisa et al.	Botswana			✓					✓			✓	✓	✓	✓	✓	✓	✓	✓	✓	✓	✓	✓	
24. Getting to the roots of evaluation capacity building in the Global South: multiple streams model to frame the agenda status of evaluation in turkey	Peer reviewed Journal Article	2016	Cakici et al.	Ethiopia	✓						✓		✓	✓	✓		✓	✓		✓	✓	✓	✓				
25. Sexy carnival on the powwow trail: HIV Prevention by and for Indigenous Youth	Peer reviewed Journal Article	2016	Monchalin et al.	Canada					✓						✓		✓			✓	✓	✓					
26. Lessons on decolonising evaluation from Kaupapa Māori Evaluation	Peer reviewed Journal Article	2016	Cram, F.	New Zealand		✓										✓	✓	✓	✓	✓	✓	✓	✓	✓	✓		
27. Negotiating solidarity between indigenous and transformative paradigms in evaluation	Peer reviewed Journal Article	2016	Cram et al.	New Zealand				✓																			
28. Kaupapa Māori evaluation: A collaborative journey	Peer reviewed Journal Article	2017	Carlson et al.	New Zealand				✓						✓			✓	✓	✓	✓	✓	✓	✓	✓	✓		
29. How do Masters of Public Health programs teach monitoring and evaluation?	Peer reviewed Journal Article	2017	Negandhi et al.	India												✓	✓	✓				✓					
30. Critical evaluation of international health programs: Reframing global health and evaluation	Peer reviewed Journal Article	2017	Chi et al.	USA & Chile						✓				✓		✓	✓		✓	✓	✓	✓		✓	✓		
31. Looking backward but moving forward: honouring the sacred and asserting the sovereign in Indigenous evaluation	Peer reviewed Journal Article	2018	Bowman-Farrell, N.	USA				✓									✓	✓	✓	✓	✓	✓	✓	✓	✓		✓
32. Conceptualizing evaluations in African contexts	Peer reviewed Journal Article	2018	Gaotlhobogwe et al.	Botswana		✓						✓					✓	✓	✓		✓	✓	✓			✓	
33. Body map storytelling as a health research methodology: blurred lines creating clear pictures	Peer reviewed Journal Article	2018	Gastaldo et al.	Spain				✓								✓	✓		✓	✓							✓
34. A new look at impact evaluation capacity in Sub-Saharan Africa	Research brief	2019	Altshuler et al.	South Africa						✓				✓		✓	✓	✓									
35. ‘We were made to feel comfortable and … safe’: co-creating, delivering, and evaluating coach education and health promotion workshops with Aboriginal Australian peoples	Peer reviewed Journal Article	2019	Bennie et al.	Australia		✓					✓		✓	✓			✓	✓	✓	✓	✓	✓	✓	✓	✓		✓
36. Towards postcolonial capacity building methodologies–some remarks on the experiences of health researchers from Mozambique and Angola	Peer reviewed Journal Article	2019	Carvalho et al.	Portugal			✓									✓	✓	✓			✓						
37. Examining indigenous food sovereignty as a conceptual framework	Peer reviewed Journal Article	2019	Ray et al.	Canada		✓											✓				✓		✓	✓			
38. White privilege and the decolonization work needed in evaluation to support indigenous sovereignty and self-determination	Peer reviewed Journal Article	2019	McKegg, K.	New Zealand		✓								✓		✓	✓	✓	✓	✓	✓	✓		✓	✓		
39. Decolonizing epidemics	Dissertation	2019	Deane Ferguson, E.	USA	✓											✓					✓						✓
40. Ukombozi means liberation: A case for decolonizing global health research, methodology, and praxis	Thesis	2019	Millet, H.	USA						✓				✓		✓	✓	✓	✓	✓	✓	✓	✓	✓			
41. #DecolonizeGlobalHealth: Rewriting the narrative of global health	Blog post	2019	Guinto, R.	Philippines	✓							✓		✓		✓				✓	✓					✓	
42. The foreign gaze: authorship in academic global health	Editorial	2019	Abimbola, S.	Australia		✓										✓			✓	✓	✓						
43. On the coloniality of global public health	Think piece	2019	Richardson, E.	USA	✓							✓		✓		✓	✓	✓	✓	✓	✓	✓				✓	✓
44. Pandemicity, COVID-19, and the limits of public health science	Commentary	2020	Richardson, E.	USA	✓									✓	✓	✓	✓		✓	✓	✓			✓	✓		✓
45. Indigenous health service evaluation	Peer reviewed Journal Article	2020	Firestone et al.	Canada		✓								✓	✓		✓	✓	✓	✓		✓	✓	✓	✓		
46. Integrative and complementary practices in the health field: towards a decolonization of knowledge and practices	Peer reviewed Journal Article	2020	Guimarães et al.	Brazil & Portugal				✓				✓		✓		✓	✓	✓			✓	✓	✓				
47. Mystic medicine: Afro-Jamaican religio-cultural epistemology and the decolonization of health	Thesis	2020	Wumkes, J.	USA		✓						✓				✓						✓		✓	✓	✓	
48. Decolonizing global public health	Commentary	2020	Affun-Adegbulu et al.	Belgium & UK	✓											✓	✓	✓									
49. Solidarity in global health research	Peer reviewed Journal Article	2020	Daftary et al.	Canada & South Africa	✓									✓		✓	✓	✓	✓	✓	✓	✓	✓				
50. Global health beyond geographical boundaries: reflections from global health education	Commentary	2020	Van Wees et al.	Sweden & USA					✓							✓	✓	✓			✓						
51. Decolonizing global public health: if not now then when?	Commentary	2020	Büyüm et al.	USA	✓									✓		✓	✓		✓		✓						
52. Decolonizing global health education: rethinking institutional partnerships and approaches	Commentary	2020	Eichbum et al.	USA, Tanzania & South Africa	✓							✓		✓		✓	✓	✓	✓	✓	✓	✓	✓	✓	✓	✓	
53. The C-Word: Tackling the enduring legacy of colonialism in global health	News article	2020	Saha et al.	Bangladesh		✓								✓		✓					✓						
54. Teaching global health from the south: challenges and proposals	Peer reviewed Journal Article	2020	Montenegro et al.	Chile					✓							✓	✓	✓	✓	✓	✓	✓					
55. How (not) to write about global health	Editorial	2020	Jumbam, D.	Ghana	✓									✓		✓	✓	✓	✓	✓	✓	✓	✓				
56. Bridging western and Indigenous knowledge through intercultural dialogue: lessons from participatory	Practice note	2020	Sarmiento et al.	Canada, Colombia & Mexico					✓							✓	✓	✓	✓	✓	✓	✓	✓	✓	✓	✓	✓
57. Research imperialism resurfaces in South Africa in the midst of the COVID-19 pandemic–this time, via a digital portal	Editorial	2020	Moodley, K.	South Africa	✓										✓	✓	✓	✓	✓	✓	✓		✓	✓	✓		✓

INCLUSION CRITERIA: (CL) calls for decolonization of global health or evaluation or global health evaluation; (CO) conceptualizes decolonization of global health or evaluation or global health evaluation; (DD) defines decolonization of global health or evaluation or global health evaluation; (EM) explicates methods for decolonization of global health or evaluation or global health evaluation; (DIC) develops interventions or curriculums to decolonize global health or evaluation or global health evaluation; (DCA) demonstrates capacity for decolonization of global health or evaluation or global health evaluation. THEMES: (SD) self-determination; (DM) decolonising individual minds; (IS) individual sovereignty; (RF) re-orient funders and reform funding mechanisms; (ELP) enabling legislations and policies; (CK) co-option of knowledge generation, production, mobilisation & translation (CK);(CP) co-production of curricula, methods, and theoretical frameworks (CP); (SCE) sustainable capacity exchange; (PC) prioritise community support, engagement and consent; (PG) positionality and gaze (PG); (PP) power and privilege; (ES) emphasise spiritual, holistic, cultural, safety and liberty; (AM) adopting mutually beneficial reciprocity; (CSD) collective self-determination; (CS) collective sovereignty; (DCM) decolonising collective minds; (RR) repairing and regaining trust against a historical backdrop of mistrust

**Fig 2:**
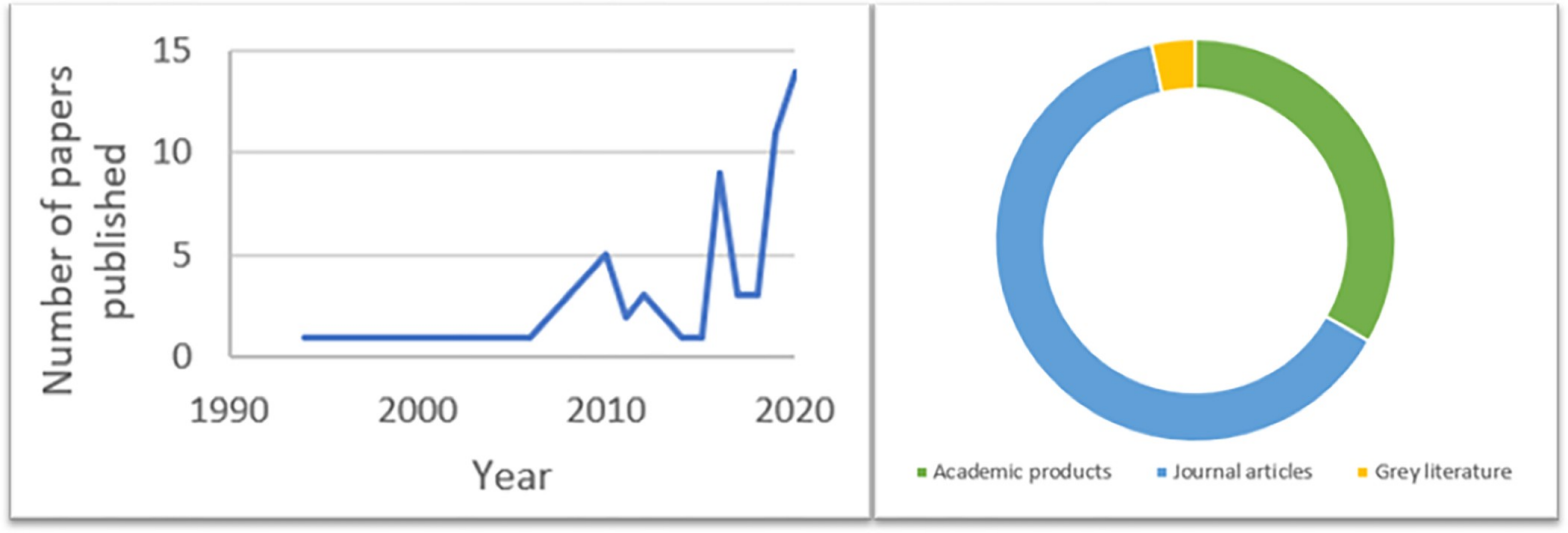
Number and types of papers included in our review published between 1994–2020.

**Fig 3:**
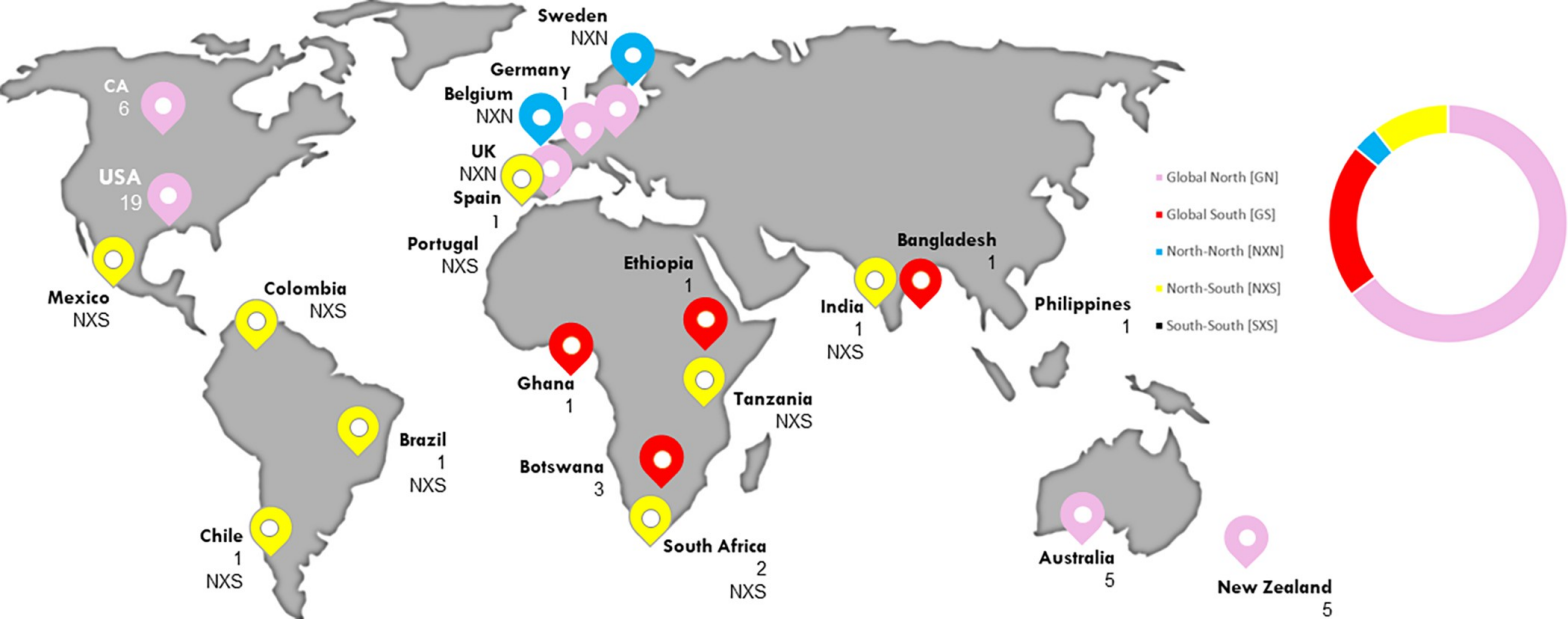
Countries and total number of papers associated with geographical regions.

One researcher subsequently computed frequencies of records published on each topic by publication year, publication type, and geographic location. Additionally, collaboratively written records were coded into three categories: North X North collaboration, defined as a collaborative effort in which all authors were affiliated with an institution located in the Global North; North X South collaboration, defined as a collaborative effort in which one or more authors affiliated with an institution located in the Global North collaborated with at least one co-author affiliated with an institution located in the Global South or vice-versa; and South X South collaboration, defined as a collaborative effort in which all authors were affiliated with an institution located in the Global South. Figs 2 and 3 and Tab 2 present these frequencies along with the factors present within each article included in our study respectively. As a final step, a member of the research team conducted two rounds of thematic analysis of the summarised results, with feedback from the entire research team. Preliminary findings were drafted in narrative and visual form.

### Statement on researcher positionality, reflexivity, and gaze

As noted above with respect to the authors of records included in our study, positionality is layered, fluid, intersectional, and sometimes conflicting rather than unidimensional, fixed, or static even within an individual author, let alone within a team. While our own authorship team members all share the experience of settler or franchise colonization and colonial educations—reflected in part by our study’s delimitation to the colonial language of English—we diverge with respect to classifications of gender, religion, class, and caste (as applicable), among other dimensions of difference—both those of our upbringings and those of our current situations. Some are still experiencing occupation or minoritisation. Others may enjoy political independence or numerical majority status, even as they live with the ongoing legacy of colonization and neo-colonization. Some represent the first generation in their family to gain access to internationally-recognized educational credentials. Others continue to benefit from the intergenerationally compounded access and opportunity that arises from their ancestral community or caste’s socio-economic and spiritual exclusion, exploitation, and violation of other groups, which colonial powers often take advantage of and exacerbate, even as they experience the effects of the racialized and gendered economy of global health and evaluation. These nuances and tensions complicate any uniformly authentic or subaltern voice that we may want or try to claim, and that others may attribute to us based on the geographic location of our institutional affiliations.

These nuances and tensions further complicate our own gaze, as our colonial educations derive from European languages and knowledge systems that were refined during, and for purposes of, imperial and capitalist expansion. As tied as we may be to our ancestral systems of knowledge and values, the lens through which we interpret the experiences of those we consider our people is inevitably colonially influenced [35]. This manifests in our struggle to name patterns that global health evaluation widely observes—even if it disavows—in ways that are accurate and that acknowledge both the experience of victimisation from structures of exclusion, marginalisation, infantilisation, and violence [35] as well as the individual and collective agency involved in survival and resistance. Additionally, the colonial gaze is evident in the choices that we made regarding capitalisation, terminology, and comparisons as part of our analysis and reporting. Examples include decisions about whether and when to capitalise the first letter of words like “indigenous”: When do we do so intentionally to honour diasporic political identity and solidarity, and when do we do so reluctantly to facilitate legibility and recognisability for a disproportionately white, colonially educated readership who is accustomed to artificial, racialized categories? The latter amplifies the ongoing flattening of within-group difference—the essentialisation of analytical categories—at the expense of more specific references to sovereign peoples or ancestral names for culturally, politically, or linguistically distinct groups or micro-nations. Third, as raised earlier, the lens of our gaze shows up in our tendency to conflate culture, nationality, and geography. Fourth, the gaze materialises in our distinction and potential otherisation of ways of knowing that are indigenous to the Americas, Asia, and Africa, including South/ West/ Central Asia and North Africa, as well as even Europe itself—as inherently spiritual and relational. Those of us schooled in colonial academic traditions often fail to acknowledge how similar forces also shaped and continue to shape contemporary knowledge systems derived from the court-supported and church-supported European Enlightenment, which was under-girded by religious doctrine and state power. Shaping of the latter, however, continues to happen in ways that scientific and positivistic narratives of rationality and individual merit shroud.

Lastly and perhaps most importantly, micro level internalisation of the colonial gaze is reflected in our use of terminology that relies on dichotomies like “First World/ Third World,” “developed/ developing,” “Global South/ Global North,” “Eastern/ Western,” “insider/ outsider/ insider-outsider,” “low-income/ high income,” as well as “power and privilege,” all of which heighten between-group difference. The dialectical nature of identity development notwithstanding, these dichotomous constructions of categorical difference treat “have nots” and “haves” as pre-social, fixed, and presumably mutually exclusive groups. This construction of difference contrasts with one which acknowledges how groups are continuously differentiated from each other—and from whiteness—through socio-economic processes, namely, the asymmetrically structured exchange of multiple types of capital, resources, power, or energy more generally. With respect to global health evaluation, neither “North/ South” nor “East/ West” maps onto hemispheric boundaries, after all, but both correspond closely to patterns of colonisation and racialisation. In a similar way, “high-/ low-income” and “power and privilege” fail to account for the source of income, power, and privilege, and direction of their flow, wherein exploitative, extractive relations systematically devalue the labour, land, and knowledge of many individual and collective bodies even as they produce great value for a few other individual and collective bodies. Migration patterns and brain-drain heavily influenced by colonisation and enslavement further complicate these categorical distinctions—especially “insider/ outsider/ insider-outsider” [36]. Referring to them in ways that obscure the material conditions and socio-economic relations that continuously (re)produce the observed difference allows such relations to persist, unchecked.

## Results

This section provides a descriptive summary of the research sample, summarizes how the advocates of the decolonisation of global health evaluation describe the decolonisation of global health evaluation, and adapts the socio-ecological framework to categorize the factors that sampled records associated with the decolonisation of global health evaluation. Within each level, factors are organized by theme, and their implications are elaborated upon in the Discussion section.

### Descriptive summary of sampled papers

In total, 57 records, ranging in publication date from 1994 to 2020, were selected and charted for this review. Table 2 details descriptive characteristics, inclusion criteria, and factors coded for the reviewed records. Records published on the decolonisation of global health, of evaluation, or of global health evaluation have steadily increased over time, reaching their peak in 2020. The majority of reviewed records were peer-reviewed journal articles (63.16%) followed by peer-reviewed or non-peer reviewed academic documents (33.33%) such as book chapters, commentaries, research briefs, reports, seminar papers, dissertations, editorials, etc. A small percentage of reviewed papers consisted of grey literature such as blog posts and news articles (Fig 2). Authors reporting institutional affiliations in the Global North published the majority of the reviewed papers (65.0%), with just a handful of reviewed papers reporting authors from African (12.28%), Asian (5.26%), or South American (3.51%) countries, or a North-South collaboration (10.53%; Fig 3) [37].

### RQ1. How do advocates of the decolonisation of global health evaluation describe the decolonisation of global health evaluation?

**Fig 4:**
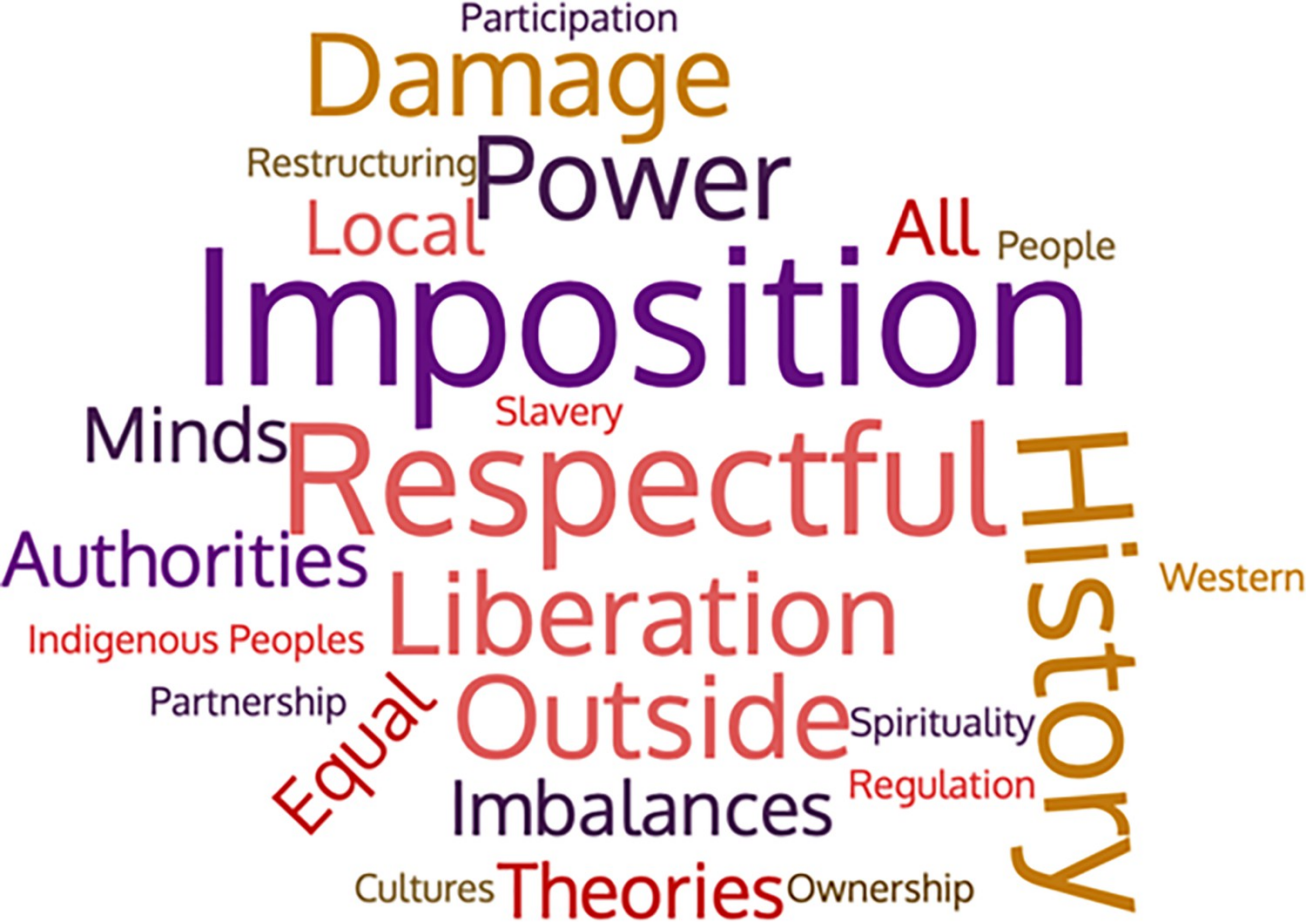
Word cloud of definitions extracted from sampled records.

To derive an understanding of how the decolonisation of global health evaluation movement advocates describe decolonisation of global health evaluation, we relied on sampled records which included definitions as an inclusion criterion (8.7% or 5 out of 57 records) as well as the summary results documents prepared by research team members with a sub-section collating definitions provided within their assigned group of records. A research team member identified key words (e.g., “cultures,” “slavery,” “colonial”) and phrases from the definitions of global health evaluation that these two sources offered (See Fig 4 for a visual representation of recurring words and phrases associated with definitions of the decolonisation of global health evaluation in the reviewed records). Based on a thematic analysis of definitions for the decolonisation of global health evaluation available and descriptions listed within sampled records, advocates of the decolonisation of global health evaluation describe it as:

*A transformative and liberatory movement that considers the effects of imperialism, slavery, racism, and colonialism on directly or indirectly colonised populations, and aims to restructure power imbalances within the fields of global health and evaluation to establish equitable, mutually beneficial, and reciprocal partnerships between those who continue to profit from the above forces of oppression and those who continue to lose from them*.

### RQ2. What socio-ecological factors do advocates of the decolonisation of global health evaluation associate with the decolonisation of global health evaluation?

**Fig 5:**
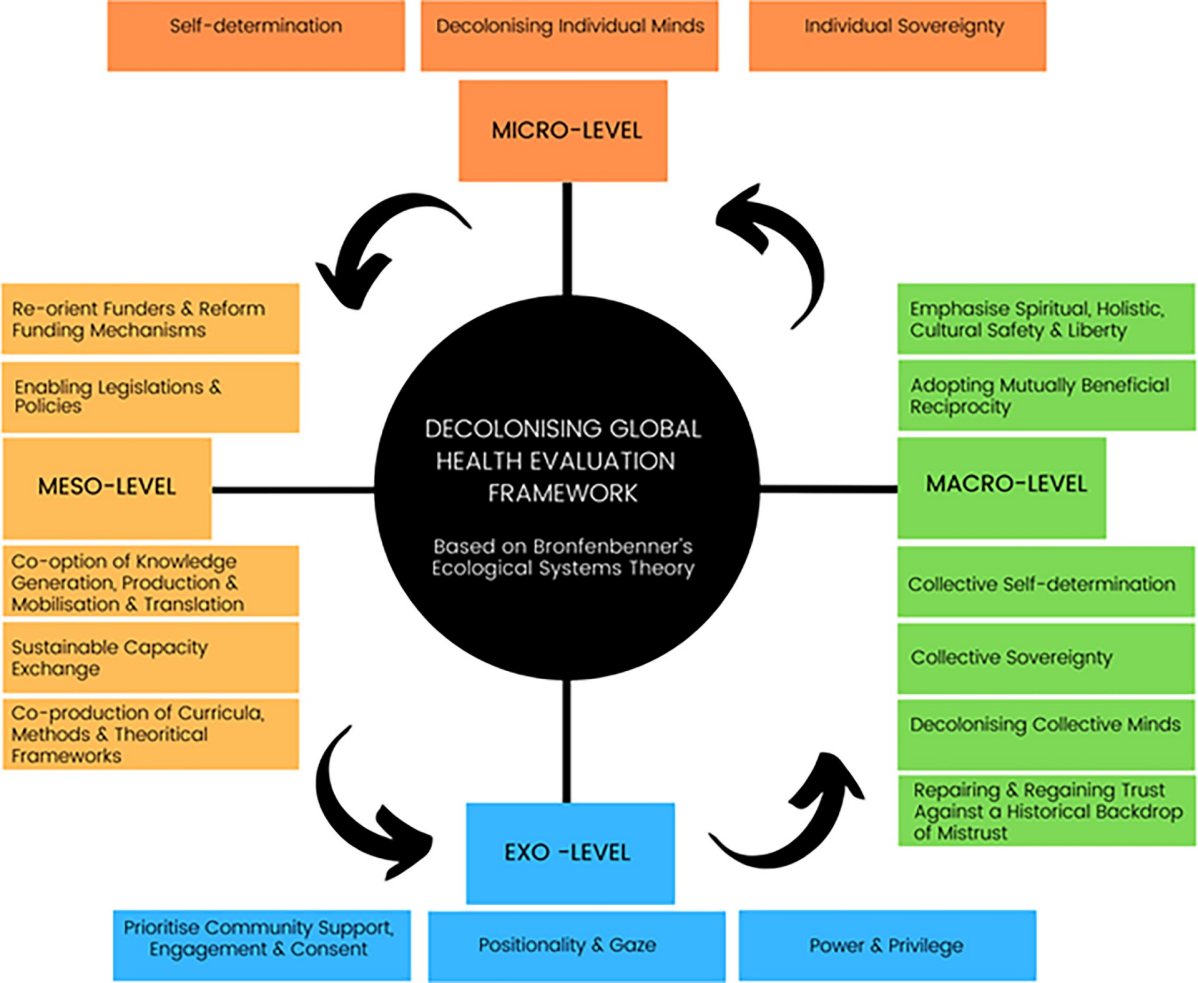
Socio-ecological factors associated with decolonisation of global health evaluation.

Following the scoping review, we used Bronfenbrenner’s socio-ecological model [31] to identify, co-develop, and organise themes (Tab 2). Factors that influence decolonising global health evaluation include micro level themes (individuals’ and groups’ perceptions, views, stances, and attitudes); meso level themes (largely related to community, organisations, and systems); exo level themes (mainly national); and macro level themes (including collective cultural values and wider economic conditions) (Fig 5). Analyses of sub-themes are grouped under each level below. All reviewed records included factors from multiple, and typically at least three, levels of the Bronfenbrenner’s model as opposed to just one. Reviewed records tended to emphasize the meso and exo levels relative to the micro level, in particular.

### Micro level

According to advocates of the decolonisation of global health evaluation movement, *at the micro-level*, the decolonisation of global health evaluation involves self-determination of individuals, sovereignty of individuals, and decolonisation of individual minds. 14.03% or 8 out of 57 sampled records associated these micro-level factors as being integral to the decolonisation of global health evaluation (Refer to Table 2 for a listing of these records).

*Self-determination and sovereignty of individuals*. Sarmiento and colleagues (2020) [6] denote how the fields of global health and evaluation currently adopt a deficit-based and damage-centred mindset, projecting native and non-western spheres and individuals as at-risk, vulnerable, unaware, and lacking knowledge to function with sovereignty and self-determination [6]. Self-determination of social problems and solutions by local individuals contests this hegemony of oppressive physical or mental constructs that default to external saviours introducing reform and development, resisting proxy colonising forces’ direct and indirect structural influence. As Abimbola (2018) [38] explains:

*We can begin to truly decolonise global health by being aware of what we do not know, that people understand their own lives better than we could ever do*, *that they*

*And only they can truly improve their own circumstances and that those of us who work in global health are only, at best, enablers [38]*.

Robertson and colleagues (2004) [39] advise that when recipients of global health evaluation efforts are empowered and sovereign at the individual level, they are no longer performing the role of passive objects of research and interventions. Rather, individuals with self-determination and sovereignty are self-actualised, active participants and contributors to global health evaluation efforts—generating data, interpreting data, and shaping dissemination and translation of outputs for community needs and transformation. Respecting and acknowledging the self-determination and sovereignty of the recipients of global health evaluation efforts requires global health evaluation professionals to reframe their mental models first and foremost. It requires them to be intentional in allowing the recipients to lead efforts and to serve as followers recognizing their own privilege, power, positionality and gaze to counter racist an oppressive mindsets and actions [40] Several advocates of the decolonisation of global health evaluation successfully demonstrate these principles in practice. Bennie and colleagues (2019) [41] applied the four domains of an Aboriginal Ngaa-bi-nya health and social evaluation framework to co-create and deliver a “culturally respectful, meaningful, and beneficial health education and promotion coaching workshop program with, and for, Aboriginal peoples” [41]. Similarly, Flicker and colleagues (2014) [42] co-designed and co-implemented an arts-based research approach to collaboratively respond to rising rates of HIV within Indigenous communities with community-based researchers and Indigenous youth. Their approach was intentional in affirming and building upon the sovereignty and self-determination of Indigenous youth by adopting a strengths and resilience-based mindset while acknowledging historical colonial and structural oppression. Indigenous youth invested in the arts-based research program shared that they felt empowered to be “living in this world too” [42] with self-determination and sovereignty.

Advocates of the decolonisation of global health evaluation movement cite concerns that in the absence of such intentional decolonisation efforts, deliverers and recipients of global health evaluation efforts may not consciously awaken to the beneficial impact derived from affirming and asserting the sovereignty and self-determination of individuals [11] For recipients and deliverers of global health evaluation efforts to consciously awaken to these realities, they require an internal paradigm shift to transform one’s minds and critically reflect on current norms and practices within the fields of global health and evaluation state scholars such as Büyüm (2020) [14], Guimarães (2020) [43] and Eichbaum (2020) [44].

*Decolonisation of individual minds*. Richardson et al. (2020) [11] and Sarmiento et al. (2020) [6] elaborate on how colonialism destroys peoples’ economies, cultures, values, religion, and much more—unravelling the social fabric and safety net of indigenous societies. Colonised peoples subsequently often doubt their own abilities and competencies, distrusting and undervaluing the ability of their nations to define the way that governance, global health, or evaluation should be conceptualised and approached [11].

*Many Aboriginal people are suffering not simply from specific diseases and social problems, but also from a depression of spirit resulting from 200 or more years of damage to their cultures, languages, identities and self-respect [45]*.

The decolonisation of individual minds refers to repairing and reversing those internalised effects of colonization—specifically addressing how the internalisation of colonial mindsets affects global health partnerships and influences decision making and policy formulation [11, 44]. Decolonisation systematically liberates colonised minds and ways of thinking, culturally, politically, and mentally [46]. Decolonisation of the self must therefore facilitate critical examination of mental and normative models through creative and therapeutic arts-based approaches [41] or religious and spiritual mediums^42^ which facilitate a shift in “how individuals relate to the world, respond to their internal desires, and as a result, transform into a more holistic version of themselves” [14, 41].

### Meso level

*At the meso-level*, advocates of the decolonisation of global health evaluation movement recommend reorienting funders and reforming funding mechanisms; enabling legislation and policies; resisting the co-option of knowledge generation, production, translation, and mobilisation; investing in sustainable capacity exchange; and engaging in the co-production of curricula, methods, and theoretical frameworks. 94.73% or 54 of 57 sampled records associated these meso-level factors as being integral to the decolonisation of global health evaluation (Refer to Table 2 for a listing of these records).

*Reorientation of funders and reform of funding mechanisms*. Decolonisation of the global health evaluation movement advocates consistently pose the question: is it possible to decolonise global health evaluation if “he who pays the piper calls the tune,” and funders and funding institutions disproportionately represent western countries and institutions? [6, 39, 47–49] The preponderance of external funding, specifically from former colonial powers, means that funding agendas and streams typically reflect imperial interests [see Levich (2015) [50] and Waitzkin & Jasso-Aguilar (2015) [51] for a definition of imperialism and explication of its historical and continuing influence on global health evaluation]. Several scholars such as Chi (2016) [21], Hopson (2012) [47], Sarmiento (2020) [6] and Robertson (2004) [39] highlight how programming is formulated based on inflexible funds, fixed timelines and shifts in the internal dynamics of donors—centring their priorities, often to the detriment of local communities. Chilisa (2015) [48] and Horton (2019) [49] caution that a skewed source of funding constitutes a hazard to global health evaluation because foreign governments, multi-lateral agencies, and international non-profit/non-governmental organizations make decisions about what to fund and how to deploy funding—at best based on national, regional, or supra-national economic and developmental goals—with little or no input from the groups on whom the global health and evaluation efforts would be directed [49]. Lessons on how to veer away from dependence on external funding and international aid (e.g., how to increase individual and collective self-determination and sovereignty; how to enhance capacity to pivot, increase, and leverage local financial, technical, and other resource pools) can be drawn from countries now transitioning from low to middle-income status, which render them ineligible to receive donor aid [52].

*Enabling legislation and policies*. Decolonised global health interventions and evaluations prioritize the host country, local authorities, and stakeholders in addition to respecting local cultures, values, laws, and sovereignty assert Saha and colleagues (2019) [7]. While it the current norm for global health interventions and evaluations to require prior Institutional Board Reviews or ethical clearances, reviews and clearances from foreign schools and governments alone are insufficient. Beyond engaging with and abiding by appropriate ethical bodies and codes of ethics, decolonisation involves scrutinizing legislation and policies, replacing them as necessary with those that enable local control. Failure to prioritise local or indigenous languages and cultural and social contexts during policy making and enforcement presents a barrier to health care for local and indigenous populations, actively harming them.

In Guatemala, for example, participation in research trials or community-based global health often requires fluency in Spanish, despite national-level policies stating that health care must be accessible in ones’ language of choice according to Flood and Rohloff (2018) [53]. Similarly, Sarmiento (2020) [6] denotes that despite a preference among expectant mothers in Mexico for indigenous birthing rituals, practices, and traditional midwifery—especially in rural settings with few skilled healthcare providers—local health systems instated sanctions on those who chose these rather than institutionalised hospital births, denying them access to birth registration and state-provided child support. In the process, they displaced the knowledge, leadership, and livelihood of indigenous midwives, ultimately harming pregnant indigenous women. Without services in their language and providers familiar with their traditions during delivery, they were unable to choose and advocate for indigenous birthing positions. Being caught between two health systems that are at odds with each other increased their risk for birth complications.

*Co-option of knowledge generation*, *production*, *translation*, *and mobilisation*. Colonisation not only occurs by hijacking and debilitating spirits and minds, enforcing pro-colonial legislation that actively oppresses, and policy-making that erases, regulates, or criminalizes local cultures, languages, and norms [42]. It is thought provoking that our results point to scholars in the Global North domineering presence as advocates for the decolonisation of global health evaluation movement. This points to the potential co-option or take-over of the movement where exclusively Global North scholars have a dominant presence and voice while exclusively Global South scholars appear to be entirely absent. Colonisation in global health evaluation has perhaps always occurred insidiously, such as through “safari” and “helicopter” initiatives that are tourist products masked as practicums for students in Europe and European settler states. Van Wees and colleagues (2020) [54] raise objections to the framing global health as health problems that take place “somewhere else but specifically in low-income countries,” global health programs and curricula offer trips to the Global South for global health missions. Caciki (2016) [55] and Smith (1999) [56] exemplify this predilection with the quote below:

*This overall essentialist tone in Western research and its cognate field of evaluation: Research “through imperial eyes” describes an approach which assumes that Western ideas about the most fundamental things are the only ideas possible to hold, certainly the only rational ideas, and the only ideas which can make sense of the world, of reality, of social life, and of human beings. It is an approach to Indigenous peoples which still conveys a sense of innate superiority and an overabundance of desire to bring progress into the lives of Indigenous peoples—spiritually, intellectually, socially, and economically [55, 56]*.

Because such engagements operate for a limited period and engage minimally with local leaders and structures, they offer the communities whose data they extract and lives they entangle with little that is of substantial use value or that is sustainable. Instead, they offer participating institutions and individuals’ opportunities for their own advancement and edification [39]. Entrenched in neo-colonial mindsets, such practices wield a heavy price in terms of their carbon, economic, and energy footprint.

Global health and evaluation programs compound such asymmetries in knowledge production, generation, and mobilisation by generally taking place in English and other colonial languages, particularly of Europe. International journals disproportionately publish scholarship in English and other colonial languages [57, 58] and ostracize publications in local or predatory journals, which many local researchers resort to because of the exclusive and exclusionary practices of high-impact journalsc [1, 58]. Global health evaluation professionals in high-income countries lead, manage, and attend academic and organisational centres that are headquartered in high-income countries. They hold key conferences with fees, travel costs, and visa restrictions that are inaccessible for the majority of the Global South [59]. The circulation of ideas and citations among a global minority steeped in colonial knowledge systems has contributed to fixed epistemological positions that centre the foreign or white gaze within which “a certain global distribution has consolidated expertise (in the ‘north’) and need (in the ‘south’)” [21, 60].

*Sustainable exchange of capacity*. The United Nation’s Sustainable Development Goals (SDG 17) embed capacity building through North-South and South-South cooperation as a strategic priority for research partnerships in science and technology to foster innovation [23, 57]. Yet such cooperative exchange mostly remains performative and lopsided, with North-South paradigmatic, academic, and experiential rifts fuelled by historical and structural co-option of knowledge generation, production, and mobilisation and with conflation of research partnerships and capacity building efforts [55, 57]. Advocates of the decolonisation of global health evaluation movement such as Carvalho (2019) [57] and Hudib (2016) [61] recommend the following as potential pathways toward establishing sustainable capacity exchange: adopting cross-cultural humility [57]; suspending epistemological authority [61]; and developing symmetric methodologies [61], fair access to technologies and resources [61], and systems to exchange (as opposed to build) capacity [61]. Learning and growth for all parties is both the goal and outcome of capacity exchange.

*Co-production of curricula*, *methods*, *and theoretical frameworks*. Local communities’ priorities, principles, and methods guide frameworks in decolonised global health evaluation. A randomised control trial conducted by Sarmiento and colleagues [6] in Mexico to examine the effects of inter-cultural dialogue on maternal health highlights the potential benefit of engaging in co-production with local communities as well as the potential harm that can result from not doing so. This study found that birth complications significantly decreased among indigenous women in the treatment arm compared to the control arm. It also demonstrates the effects of more than ten years of co-production alongside the local community.

The responsibility for co-producing curricula, methods, and theoretical frameworks rests squarely on those seeking to engage with communities whose cultural and historical heritages continue to be subjugated. Among the Māori of New Zealand, *tikanga*—which refers to customary practices or behaviours—decrees that evaluation is only authoritative when practitioners sustain *Kawa whakaruruhau* (cultural safety and appropriateness) in all stages of the research [22]. Similarly, in the African context, Chilisa and colleagues suggest that the ideal community development evaluation framework is based on five interrelated and complementary principles rooted in *ubuntu* [48]. While they propose that such a relational framework is indigenous to and pervasive across all of Africa, others caution against totalising and potentially essentialising characterizations of a monolithic or universal “African” culture or society [see Ram and Affun-Adebulu (2020) [62] for a more nuanced take on this matter].

### Exo level

*At the exo level*, decolonisation of global health evaluation prioritises community support, consent, and engagement or involvement in addition to the cultivation of collective awareness and understanding of the micro level internalisation of power and privilege, positionality, and gaze [1, 22]. 84.21% or 48 out of 57 sampled records associated these exo-level factors as being integral to the decolonisation of global health evaluation (Refer to Table 2 for a listing of these records).

*Prioritising community support*, *engagement*, *and consent*. Any sincere effort to decolonise global health evaluation garners community support, involvement, and consent as demonstrated by the Oglala Sioux CIRCLE Project evaluation researchers [39]. This principle was upheld from conceptualisation through design and implementation. Pledges followed by action demonstrated that data offered by the community for research and evaluation purposes can be used responsibly and shared transparently with the community; both to avoid exploiting them and to equip them with critical information that can empower them to successfully navigate and transform the systems and stakeholders they engage with in their day-to-day lives. This project highlights how to avoid extractive relations and decentre global health evaluators’ intentions by centering local communities. Though such depth of community participation can signal altruistic intentions, asymmetrical power dynamics at institutional and structural, even if not individual, levels can lead participatory approaches to play out as a new form of tyranny (see Dorman edited by Cooke and Kothari 2001 [63]). Decolonizing global health evaluation distinguishes between participation and collaboration. It involves collaborating with local communities as equals—as self-determined, sovereign agents of change who are capable of prioritising and strategizing [39]—beyond simply creating opportunities for them to participate within structures established by outsiders.

*Power and privilege*. The leadership of global health organisations is concentrated among those classified as white and as men, who are products of and still tied to elite institutions within the Global North, reflecting the hegemony of the field [24]. The 2020 *Global Health 50/50* report shows that high-income countries make up only 17% of the global population but are responsible for 83% of global health organisations; 50% of such organisations are based in the U.S.A. and U.K [26]. Control over more than three-quarters of the industry by a single, small group portends an unrestrained ability to wield power and unearned privilege to decide what merits evaluation, who should conduct it, and how. Decolonising global health evaluation requires shared decision-making power between the Global North and Global South. Balanced leadership can provide the necessary check on the preponderance of racist and colonial ideologies [14].

*Positionality and gaze*. Positionality is an inherently relational term. As opposed to the idea of *coming to be in relation* with, relationality could be considered the original state of being and development of personal and collective identity, like many indigenous knowledge and value systems proffer, because all human beings *begin life in relation*, in utero and then as infants still attached to their mothers [64]. Positionality’s inherently relational nature lies in its reference to the researcher’s location *in relation to* the subject matter, populations, and context [1]. Additionally, however, positionality is an inherently structural term—unlike essentialised notions of culture—that draws from standpoint theory in that it requires that researchers situate themselves in space and time—not just geographically but also historically and in terms of social location, including economically, politically, linguistically, spiritually, etc. Researcher positionality does not exist on a bipolar continuum as “insider, outsider, insider-outsider” might suggest [1, 22]. Rather, it is multidimensional, because researchers are human beings and human beings are multidimensional. Their identities are formed in part through the systems of oppression that human beings have constructed and continue to navigate.

Gaze is the viewpoint of the knowledge producer, which is inevitably shaped by their positionality. The knowledge producer’s orientation to and perspective on the subject matter, population, and context affect every phase of the evaluation process [22]. Gaze is largely not a matter of individual choice, but the biases inherent in any individual gaze can be balanced by mitigating the influence of individuals in the evaluation process through the relational, collaborative production of knowledge. The “insider, outsider, insider-outsider” construction of positionality and gaze are themselves reductive if not false in that even locals—certainly those with global health evaluation and decision-making power—likely gained their internationally recognisable understanding of and credentials in global health evaluation through a foreign language and curriculum, even if that foreignness is defined by classifications and experiences of caste, class, or spirituality in addition to or instead of geography or race.

Still, Cram (2016) [22] emphasises the positionality (whether outsider, insider, outsider-insider or multidimensional viewpoint is rooted in social and economic location ultimately influencing relationality) adopted by a researcher affects all phases of research and evaluation efforts. Being blind to ones’ positionality and gaze can lead to adverse outcomes has been detailed by Moodley (2020) [65]. University of Kent researchers fielded a survey seeking to research views of South African healthcare practitioners on abortion access and provision of services across a variety of sectors without local ethical approvals or meaningful local engagement. When these researchers were alerted to the absence of South African researchers on the project as well as their lack of ethical approval, their response was to publish a letter in a local medical brief titled ‘University of Kent wants South African input on abortion project’. Moodley (2020) [65] objects to the colonialist tones and raises concerns about harms produced by such research conducted exclusively with colonialist positionality and gaze without meaningful local context and input.

### Macro level

*At the macro level*, the decolonisation of global health evaluation movement advocates advise facilitating the decolonisation of global health evaluation necessitates an emphasis on holistic understandings of safety and liberation that encompass spirituality and culture. It involves acknowledging a historical backdrop of mistrust and repairing or restoring trust (noting that “repairing” and “restoring” suggest that there was trust originally). Fostering the decolonisation of global health evaluation’s collective mind—or mental model—requires articulating its collective purpose as transformative, liberatory, and focused on restructuring power imbalances within the fields of global health and evaluation to establish equitable, mutually beneficial, and reciprocal partnerships. 70.17% or 40 out of 57 sampled records associated these macro-level factors as being integral to the decolonisation of global health evaluation (Refer to Table 2 for a listing of these records).

*Emphasising safety and liberty that holistically encompass spiritual and cultural values*. Culture refers to shared meaning [66]. Culture distinguishes one community from another by embodying their history, lived experiences, knowledge, values, learned behaviours, beliefs, social hierarchies, ways of communicating, intergenerational traditions, and collective programming of the mind [67]. Sarmiento (2020) [6] and Robertson (2004) [39] underscore that aligning health with their spiritual, ancestral, and cultural values matters to all groups [6, 39]. Similarly, Iseke (2013) [68] explains:

*Decolonising and spirituality are inextricably linked: the outer and inner selves are connected through understandings of spirituality [69]*.

While spiritual beliefs and practices shape how all communities live and access knowledge, it is more explicit and perceptible among groups associated with otherized and minoritized spiritual traditions. The normativity of whiteness and Christianity, however, conceal their influence within scientism, and positivism. Colonial authorities view local traditions, cultures, and holistic health systems as backward or devilish and the people as savages suggest Sarmiento (2020) [6] and Mundel (2010) [45]. Advocates of the decolonisation of global health evaluation movement recommend global health efforts [69–71] recognise the sacred, especially in relation to health, which is tied to some of the most intimate dimensions of life. They elaborate on how value systems in Indigenous and other formerly or currently colonised populations are built around a sense of community and togetherness between the living and ancestors with guidance from elders in the community. These values are often grounded in collective and fair responsibilities, cooperation, interdependence, and interpersonal relationships among people [69–71]. And such, they recommend that decolonised global health evaluation require policy makers, funders, evaluators, and global health practitioners to embody and enact the values of local residents with whom they collaborate. For example, Wumkes (2020) [70] elaborates within their dissertation how the Jamaican biomedical healthcare system has been entirely divorced from the indigenous Afro-Jamaican healing practices resulting in a failure to serve the majority of the population. They advocate for a decolonised integrative healthcare model reconciling the differential approach to culture, spirituality, and holism adopted by biomedicine and indigenous Afro-Jamaican medicine. Along the same vein, Bowman-Farrell’s (2018) [71] Culturally Responsive Indigenous Evaluation (CRIE) evaluation model explicates how Western global health evaluation efforts can connect to and emphasise safety and liberty for cultural and spiritual values by incorporating the multidimensional “physical, mental, spiritual, and emotional” aspects of evaluation.

*Repairing and regaining trust against a historical backdrop of mistrust*. Addressing the decolonisation of global health evaluation is impossible without acknowledging the history of colonialism and its tremendous ramifications according to advocates of the decolonisation of global health evaluation movement such as Bennie (2019) [41], Bowman-Farrell (2018) [71], Horton (2019) [49] and McKegg (2019) [40] among several others. Decolonised global health explicitly must recognize and repairs the pain of the past and engages in present and future practice through the lens of social justice [40, 41, 49, 71].

*Any western medical institution more than a century old and which claims to stand for peace and justice has to confront a painful truth–that its success was built on the savage legacy of colonialism. Perhaps we deal with uncomfortable pasts by burying them, excusing them, or atoning for them. The Lancet, for example*, *is a colonial era institution [49]*

Horton’s (2019) [49] quote above acknowledges the “painful truth” of the colonial origins of global health evaluation. Some of the first schools of public health were established as part of colonial occupation and meant to protect the health of colonial authorities, the local labour and productivity that they relied upon, and their overarching imperialist aims [50, 51]. For instance, the Liverpool School of Tropical Medicine is the first school of tropical medicine in the world, having been established in November 1898. According to its website, the school’s founder was Sir Alfred Lewis, “an influential shipping magnate who made significant profits from various European countries’ colonial exploitations, mainly in Africa” [72]. The association between scientific enterprise and oppressive colonial regimes is also illustrated by rural Palestinians’ mistrust of and resistance against smallpox vaccination during British colonisation as indicated by Dadidovitch and colleagues (2007) [73]. Although we invoke historical colonisation here, colonisation continues via other agendas and means, including through the imperialistic monopolization of global health decision making and evaluation processes [refer to Levich (2015) [50] and Waitzkin & Jasso-Aguilar (2015) [51] for a thorough definition of imperialism and explication of its historical and continuing influence on global health evaluation].

*Collective self-determination and sovereignty*. The current colonial form of global health evaluation compromises collective self-determination and sovereignty and relegates local people to second-class citizenship within their own land (e.g., Palestinians in Occupied Palestinian Territories). Collective self-determination with respect to decolonised global health evaluation means that local stakeholders, people, officials, and leaders hold the power to decide how, what, and when to implement and evaluate efforts assert Cram (2016) [22], McKegg (2019) [40], Chilisa (2015) [48], Bowman-Farrell (2015) [71], Wumkes [70], among several other decolonisation of global health evaluation advocates. Similarly, collective sovereignty connotes political authority [74]. It means supreme authority within a territory over matters such as governance, policy making, resource utilisation and allocation, negotiation of borders and advocacy for inalienable rights to liberty, freedom of expression, housing, health, and human rights, etc. Collective sovereignty shapes decision making, leadership, governance, and regulations defining how to do things within a community [75].

For example, “nation building” is a term increasingly found in the literature, used particularly by leaders in Indian Country (any of the many self-governing Native American communities throughout the United States—all federal trust lands held for Native American tribes [76]). It refers to the process of constructing effective institutions of self-governance that can provide a foundation for sustainable social development, including health and education; judiciary and legal institutions; and successfully democratised political systems, advocacy, and actions. In other words, nation-building is the process of promoting individual and collective self-determination, self-governance, and sovereignty [77, 78]—ultimately improving tribal citizens’ social and economic situations through the creation of more capable, culturally legitimate institutions of governance and stewardship [39]. Translating the “nation building” ethos into praxis, advocates of the decolonisation of global health evaluation movement such as Chi and colleagues (2017) [21] challenge mainstream global health evaluation efforts while proposing the Critical International Health Program Evaluation framework which uphold the principles of collective self-determination and sovereignty. The following quote illustrates their motivations for challenging the mainstream global health evaluation efforts by developing an alternative framework to uphold collective self-determination and sovereignty during global health evaluation efforts.

*A vital tenet of our framework is that a community possesses the right to determine the path of its health development. A prerequisite of success, regardless of technical outcomes, is that programs must address communities’ high priority concerns. Current participatory methods still seldom practice community ownership of program selection because they are vulnerable to funding agencies’ predetermined priorities [21]*.

*Mutual benefit and reciprocity*. The Kaupapa Māori theory-based evaluation framework emphasises transformation as a core principle through the concept of *koha* or reciprocity [79]. Reciprocity must be inherent in all collaborative effort to facilitate collective transformation and achieve useful outcomes for the collective good [79]. Cavino (2013) [80] adds that privileging the principle of achieving mutual benefit and reciprocity in an equitable way paves the path for local communities to own and protect the knowledge that is generated. Pai (2020) [81] acknowledges the importance of centering mutual benefit and reciprocity to decolonise global health evaluation efforts. They outline ten recommendations on how global health evaluation efforts can apply the principle of mutual benefit and reciprocity which includes measuring and tracking reciprocity. Measuring and tracking reciprocity has the potential to lay bare the normatively extractive and transactional nature of global health evaluation efforts [49, 81].

*Decolonising collective minds*. The effects of colonialism are devastating and can last for generations, entrenched in colonial or neo-colonial structures and mental constructs.

*Decolonisation reaches beyond removal of colonial power and dismantling of colonial structures to include decolonisation of the mind that made the coloniser feel superior and the colonised feel inferior by enforcing structural drivers of discrimination and barriers to self-determination [32]*.

Decolonising collective minds involves removing colonial powers and conditions and dismantling colonial structures to decolonise individual and collective minds—which are mutually reinforcing [11].

*Decolonisation, therefore, is a systematic way of research and evaluation that attempts to liberate the colonized mind (individual) so that formally colonized people (collective) are not only politically emancipated, but also mentally emancipated [56]*.

Mundel (2010) [45] details that decolonising collective minds has revolutionary potential for decolonising global health evaluation efforts and society-at-large. Doing so begins with an individual paradigm shift leading to the dismantling of the dominant colonial mental models influencing the collective. This requires both the non-Indigenous and Indigenous to transform their inner selves. The non-Indigenous must then collectively mobilize to respect, engage with, and immerse in the decolonisation movement such as privileging Indigenous self-determination and sovereignty. Similarly, the Indigenous must experience an inner transformation as well such that they can collectively mobilize and advocate for decolonised global health evaluation as illustrated by Chilisa (2015) [82] with the following quote:

*Along with the African renaissance concept is the Africanisation concept which refers to ‘a process of placing the African worldview at the centre of analysis’. It can be viewed as an empowerment tool directed towards the mental decolonisation, liberation and emancipation of Africans, so that they do not see themselves only as objects of research and consumers or borrowers of knowledge, but also as producers of knowledge capable of theorising about the production of knowledge in ways embedded in the cultures and experiences of the African peoples [82]*.

## Discussion

Intended to re-imagine global health evaluation by considering its decolonisation, this study sought to define and provide a socio-ecological understanding of factors associated with the decolonisation of global health evaluation through a scoping review of papers published on the decolonisation of global health, of evaluation, or of global health evaluation. It defines the decolonisation of global health evaluation more as a means for the advocates of the movement than an end. Representing a commitment to a process—a transformative and liberatory movement that aims to restructure relations—the definition resists the product-oriented exhortations of positivism and scientism. Understanding the factors associated with decolonisation in a socio-ecological context provides practitioners and scholars in the field of global health evaluation with micro, meso, exo, and macro level mechanisms through which the experiences, perspectives, and interests of displaced and dispossessed communities can shape the evaluation of global health interventions. In the remainder of this section, we summarize the findings and interpret the meaning of our review’s results in the context of prevailing research and theory in global health evaluation; consider their implications and significance for theory and practice; and share recommendations for future practice and scholarship on its decolonisation.

### Summary & interpretation

Results of this scoping review offer global health evaluation practitioners, educators, and researchers a socio-ecological framework through which to understand the interactive, mutually-causal factors involved in decolonisation. Such an understanding allows practitioners and scholars alike to act within their spheres of influence, regardless of their personal and professional positionality or social and geographical location, with the aim of effecting change at multiple socio-ecological levels (as in micro, meso, exo, and macro). That nearly every article addressing global health, evaluation, or global health evaluation that was reviewed proferred multi-level decolonizing factors contrasts with de facto institutional understanding and approaches that rest entirely on individual level factors such as: 1) the “diversity” of individual contributors, without considering institutional and structural mechanisms that enable (or prevent) diverse perspectives to shape policy and practise; 2) the “cultural competence” of individual members of dominant groups; or 3) the benevolence of dominant institutions to “include” members of the indigenous and colonized groups whose lives their work affects [83]. Instead, the literature on decolonizing global health evaluation offers everyone engaged in global health evaluation multiple avenues to act in decolonizing ways, regardless of their identity or positional power, and the socio-ecological model articulates those avenues explicitly.

### Implications & significance

The consistency of factors identified within and across fields points to the need for intentionality among the relevant fields to act on them. In other words, while this review’s collation and organisation of decolonising factors into socio-ecological levels adds value to the relevant fields by validating the factors against each other and starting to build a conceptual framework among them—thus making them more accessible and actionable—the individual factors themselves have been documented across fields, in some cases repeatedly, for nearly 30 years, with little movement toward decolonisation as this paper defines it. The global health evaluation system’s homeostatic resistance to change and tendency toward isomorphism can be partially explained through concepts from natural and social systems that undergird the socio-ecological model employed in this paper [84]. Similarly, the system’s more specific entrenchment of inequality along pervasive, persistent, and predictable patterns can be partially explained through concepts about power offered by critical theories of systemic oppression, which this paper also employs. In the spirit of both disciplinary traditions and the results of our review, we interrogate our process and findings below.

### Strengths

This study is rare in its employment of Arksey & O’Malley’s scoping methodology [30] and in its application of Bronfenbrenner’s socio-ecological model [31] not to research subjects or program participants “out there” but rather to researchers, program staff, and program evaluators “in here”, by critically reflecting on the fields of global health and evaluation themselves. It addresses global health and evaluation separately and together, identifying factors related to decolonisation of each at multiple levels. The length and nature of the research period—during a global pandemic and international uprising with respect to multiple forms of systemic oppression including direct and indirect forms of colonisation—necessarily shaped the scope and type of engagement with the literature. Still, the overwhelming consistency and synergy across reviewed records and, indeed, fields—captured systematically through a scoping methodology and conceptual framework—allows global health evaluation to move toward a theory of transformation and an associated research agenda to support its decolonisation.

### Limitations

Our search strategy was limited to records in the English language and to a handful of databases that associated with academic and grey literature archives. Delimiting this review to English-language literature ironically re-centres the colonial powers of Europe and European settler states. Indeed, the word “decolonise” itself was coined by colonial powers, in reference to the independence of what is now the USA from Britain [85] and later in reference to independence from European colonial powers in the mid-20^th^ century [86]. Another limitation lies in its potential reification of the artificial boundaries between levels of the socio-ecological model—not unlike other artificial boundaries, between groups, that we described in our section on positionality and gaze. The micro level factors of self-determination, individual sovereignty, and decolonisation of individual minds that were identified through this review require macro and exo level interventions as much as the exo and macro level changes listed in the results require individual agency and interpersonal relationships at the micro and meso levels. The process of decolonisation of global health evaluation is thus interactive among levels and could be characterised as “inter-level” beyond simply being “multi-level.” Lastly, the results of this study reflect the literature identified through the databases specified which may produce a selection bias as it is possible we did not capture all available documents available on this topic. The study was also conducted during a global pandemic and related series of uprisings against oppression and the results have yet to be validated by practitioners or tested empirically—steps that were beyond the scope of this study but form the basis of a research agenda focused on the decolonisation of global health evaluation. Finally, with respect to positionality, the geographic location of authors’ affiliated institutions does not necessarily correspond with their individual nationality, country of current residence, upbringing, or training, nor can it be assumed to reflect any particular cultural or political identity, experience, or perspective even when it does correspond—especially when considering displaced and diasporic communities. Geographic location of authors’ affiliated institution also does not necessarily correspond with the authors’ institutional perspective, privilege, or power, especially when considering institutions serving minoritised populations in settler colonial states (such as Asian American and Native American Pacific Islander serving institutions, historically Black colleges and universities, Hispanic serving institutions, and tribal colleges). This is particularly so in cases of team authorship. Categorizing collaboratively written records by geographic location of affiliated institution only provides some insight into the system of structural opportunities, incentives, and constraints available to authors in relation to the international political economy and authors’ efforts to work within and across disparities therein.

### Recommendations

Because the publishing industry and academia privilege colonial languages and especially English, as described earlier, collaborating across language—colonial and indigenous—would mitigate but not resolve the limitations of focusing on English-language items for review. Additionally, future studies can intentionally and explicitly question and seek to further complicate taken-for-granted boundaries and associated perspectives, constructed along artificial binaries, through the research process and decisions. Finally, future research and practice can focus on the iterative application of and reflection on the factors identified through this review in various contexts. For global health evaluation to transform itself—to decolonise—it must sustain the praxis of interrogating everyday decisions and processes, asking how each channel multiple types of power: To what extent and in what ways do we continue to feed—and alternatively, do we starve—the ongoing (re)production of difference in the form of artificially constructed disparities, disproportionalities, and distinctions?

## Conclusion

In sum, decolonisation is now at the risk of becoming a comfortable, trendy but misused, exploited, performative—and most worrisome of all—commercial buzzword [7, 87, 88]. An irony Khan (2021) [69] points out is that a corresponding word does not exist within many non-European languages. Moreover, many in the Global South are unable to relate to or agree with the terminology considering its Eurocentric origin and central focus on the Global North. For some, decolonisation can only occur when colonising institutions are shuttered and cease to influence the Global South or settler-colonial contexts [7, 87]. For others, decolonisation is simply a principle under which those interested in furthering the cause can organise [7].

Our paper demonstrates that scholars and practitioners have identified a myriad of socio-ecological factors that are associated with the practice of decolonisation. We have also summarised their collective wisdom and perspectives on how to define decolonisation. Despite advancing the discourse and critical assessment of the decolonisation of global health evaluation and factors associated with the term through this review, gaps exist and obstacles remain. Echoing Khan (2015) [87], the origin of the papers we have reviewed in this study highlights how the scholarship centred on decolonising global health evaluation primarily emerges from the Global North (specifically the U.S.A., Australia, Canada, and New Zealand) (Fig 4). The ideas and knowledge we have collated thus represent the viewpoint of scholars and practitioners residing in or affiliated with institutions in these countries. We can surely concede that it is entirely feasible that the origins of many of the authors of the reviewed papers mirror the co-authors of this reviewing paper—those who have ancestral origins or national affiliation within the Global South or Indian Country but whose current or former residence or training is in the Global North. Therefore, one could argue that the gaze this paper offers is from a third perspective, between the foreign or white gaze and the local gaze, because its co-authors—and the authors of many of the reviewed papers—experience and traverse these two orthogonal spheres.

The pandemic has highlighted the social, economic, financial, and political inequities faced by the marginalised majority (82% of the world’s population)—Black, Indigenous, Peoples of Colour (BIPOC). It also lays bare a concept that was previously intangible—global interconnectedness tying together our shared destiny and threats [14]. The pandemic has also exposed and reversed the perception of the Global North’s superiority in upholding aspirational public governance, health, and well-being, as Asian and African countries effectively flattened the spread’s curve and returned to normalcy while European and North American countries continue to face the pandemic unabated during the first wave [89, 90]. On the darker side, there are now real risks of exacerbating existing apartheid (vaccine or financial) and rising inequities [91, 92]. Yet, there is also an unprecedented and urgent window of opportunity to transform and decolonise global health evaluation [14]. What might decolonisation look like under these unchartered territories? Our review offers a starting point towards framing this discussion. In revisiting whether decolonisation is a product, checklist, badge, outcome, or journey: we lean heavily towards viewing decolonisation as a journey that exacts a process of transformation towards the greater goal of achieving equity and justice.

## Supporting information

S1 Checklist Preferred Reporting Items for Systematic reviews and Meta-Analyses extension for Scoping Reviews (PRISMA-ScR) checklist. (DOCX)

S1 Table Charting framework template. (DOCX)
