# Supplementary material for: Decolonising global health evaluation: Synthesis from a scoping review
Source: PLOS Glob Public Health. 2022 Nov 16;2(11):e0000306. doi: 10.1371/journal.pgph.0000306 (PMC10021742; doi:10.1371/journal.pgph.0000306)
Supplement: S1 Table — (DOCX) [file pgph.0000306.s002.docx]

## **S1 Table. Charting framework template**

| Article title |
| --- |
| Number of pages |
| Coder |
| Publisher |
| Publication date (DD/MM/Y) |
| Type of publication |
| Authors (List in order of authorship) |
| Location of authors (city, state, country) |
| Inclusion criteria (select one of six criteria listed in the drop-down menu; if none apply select NA) |
| Definition for decolonising global health evaluation |
| Describe the methods utilized or proposed (List NA if not applicable) |
| Describe theoretical frameworks proposed (List NA if not applicable) |
| Describe barriers and facilitators |
| Describe curriculums listed (List NA if not applicable) |
| Describe role of self-determination, empowerment, and sovereignty |
| Describe role of cultural, spiritual, holistic or social justice philosophy and values |
| Describe role of funding and funders |
| Describe any legislation, policies or structural programs in place |
| Describe role of knowledge generation, mobilization, and mutually beneficial reciprocity |
| Describe any key actors and activists involved |
| Describe the role of community support, participation and consent |
| Describe the role of positionality in the community (insider, outsider, insider-outsider) |
| Describe the role of domination and control vis-à-vis liberation or transformation |
| Describe the role played by efforts to build capacity and south-to-south collaborations |
| Describe the role of trust or mistrust |
| Describe the role of solidarity |
| Describe the role of collectivism and inter-connectedness |
| Describe the role of power and privilege |
| Any additional themes unlisted in the charting framework? Please list and describe below. |
| General notes |
